# Supplementary material for: Deleterious Effects of SARS-CoV-2 Infection on Human Pancreatic Cells
Source: Front Cell Infect Microbiol. 2021 Jun 23;11:678482. doi: 10.3389/fcimb.2021.678482 (PMC8285288; doi:10.3389/fcimb.2021.678482)
Supplement: Supplementary file 1 [file DataSheet_1.pdf]

Supplemental Information

This file includes:

Supplementary Figure and Table Legends.

Supplementary Figures 1-6.

Supplementary Tables in a combined spreadsheet file containing the following supplementary tables (submitted as a separate .xlsx file).

SUPPLEMENTARY FIGURE AND TABLE LEGENDS

Supplementary Figure 1. iPanEXO organoids exhibit ACE2 expression. iPanEXO organoids exhibit polarization (E-cadherin), as well as markers of both pancreatic acinar (CTRC) and ductal cells (CK19, SOX9). These organoids exhibit ACE2 expression similar to adherent iPanEXO cultures. Scale bar represents 200 μm for large rectangle images, 50 μm for small square images, and 10 μm for zoomed panels adjacent to main images. ICC images shown here are representative results of 27 independent sites acquired. These results were pooled from 2 independent rounds of virus infections.

Supplementary Figure 2. iPanEXO ductal cells express ACE2. Day 3 post infected iPanEXO ductal cells exhibit ACE2 expression in SARS-CoV-2 infected and uninfected Mock control groups. The images below show the magnified regions of interest of DAPI and ACE2 staining in the dotted line boxes shown in the main panel for Mock and MOI 0.05 groups. Scale bar represents 200 μm. ICC images shown here are representative results of 36 independent sites acquired. These results were pooled from 2 independent rounds of virus infections.

Supplementary Figure 3. C-peptide positive cells are infected by SARS-CoV-2. iPanEXO acinar cultures contain small clusters of C-peptide protein expressing cells. A portion of these C-peptide positive cells were found to additionally express SARS-CoV-2-(S) on Day 3 (72 hours post-infection). Scale bar represents 130 μm, and 20 μm for zoomed panels adjacent to main images. ICC images shown here are representative results of 27 independent sites acquired. These results were pooled from 2 independent rounds of virus infections.

Supplementary Figure 4. Inflammatory markers were not altered by SARS-CoV-2 infection at Day 1 and as well as IL1B and TNFA at Day 3. **A.** RT-qPCR of infected and non-infected samples collected 24 hours (Day 1) after infection do not show significant change of mRNA expression of *CXCL12, IL1B, TNFA, NFKB1*, or *STAT3*. **B.** RT-qPCR of infected and non-infected samples collected 72 hours (Day 3) after infection do not show significant change of *IL1B* or *TNFA* mRNA expression. Data is shown as mean ± SEM with statistical significance determined by unpaired two-tailed t-test. RNA was extracted from aggregates of 16 biological replicates (using 96-well plate), and qPCR was run in 3 technical replicates per sample. These results were pooled from 2 independent rounds of virus infections.

Supplementary Figure 5. Post-Mortem Human Pancreas Staining of pancreatic ductal marker Cytokeratin 19. Non-COVID controls and COVID-19 patients do not exhibit SARS-CoV-2 2 Spike S1 protein (green) in pancreatic ductal population, CK19 (red). Scale bar represents 50 μM. IHC was performed in 3 non-COVID control and 3 COVID patients. The results shown here are representative 3-10 independent sites acquired per patient.

Supplementary Figure 6. mRNA expression of additional pancreatic and inflammatory markers from postmortem COVID-19 and Control patients. Post-mortem pancreatic tissues from COVID-19 patients (n=6) and non-COVID controls (n=5) were assessed for mRNA expression of **A.** endocrine markers NGN3 and INS; **B.** acinar markers CTRC, AMY1A, PTF1A and MIST1; and **C.** inflammatory markers IL1B, TNFA, CXCL12, NFKB1 and STAT3. Data is shown as mean ± SEM with statistical significance determined by unpaired two-tailed t-test. RNA was extracted from 5 non-COVID controls and 6 COVID patients. qPCR was run in 3 technical replicates per sample.

Supplementary Table 1. Formulation of base media for culture of iPSC-derived iPanEXO Acinar and Ductal cells.

Supplementary Table 2. List of primer sequences used for Real-Time qPCR.

Supplementary Table 3. Expression of Viral Transcripts in RNAseq data.

Supplementary Table 4. Significant Differentially Expressed Genes Between SARS-CoV-2 Infected and Uninfected Cultures.

Supplementary Figure 1

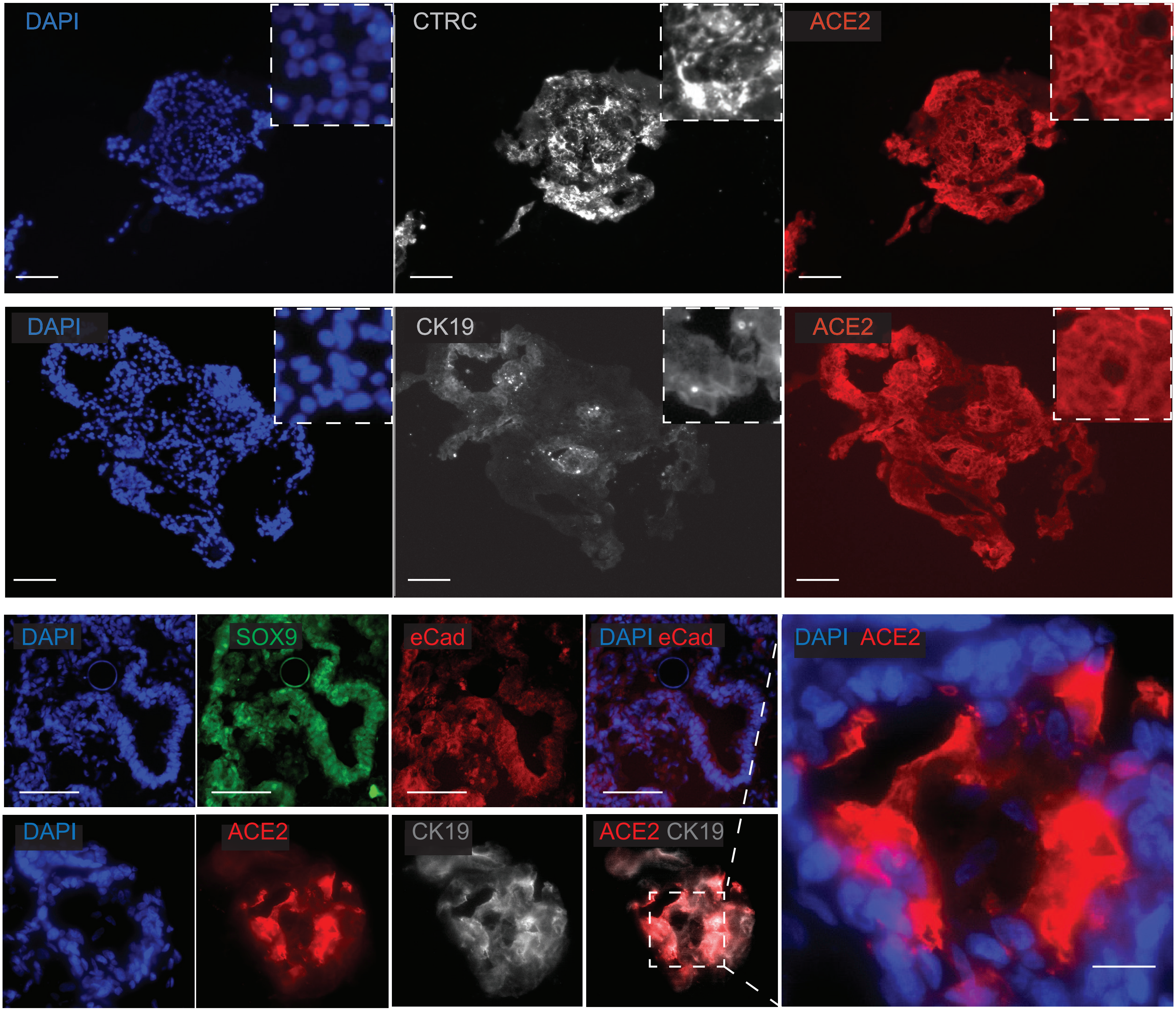

Supplementary Fig 1. iPan^{EXO} organoids exhibit ACE2 expression. iPan^{EXO} organoids exhibit polarization (E-cadherin), as well as markers of both pancreatic acinar (CTRC) and ductal cells (CK19, SOX9). These organoids exhibit ACE2 expression similar to adherent iPan^{EXO} cultures. Scale bar represents 200 μm for large rectangle images, 50 μm for small square images, and 10 μm for zoomed panels adjacent to main images. ICC images shown here are representative results of 27 independent sites acquired. These results were pooled from 2 independent rounds of virus infections.

Supplementary Figure 2

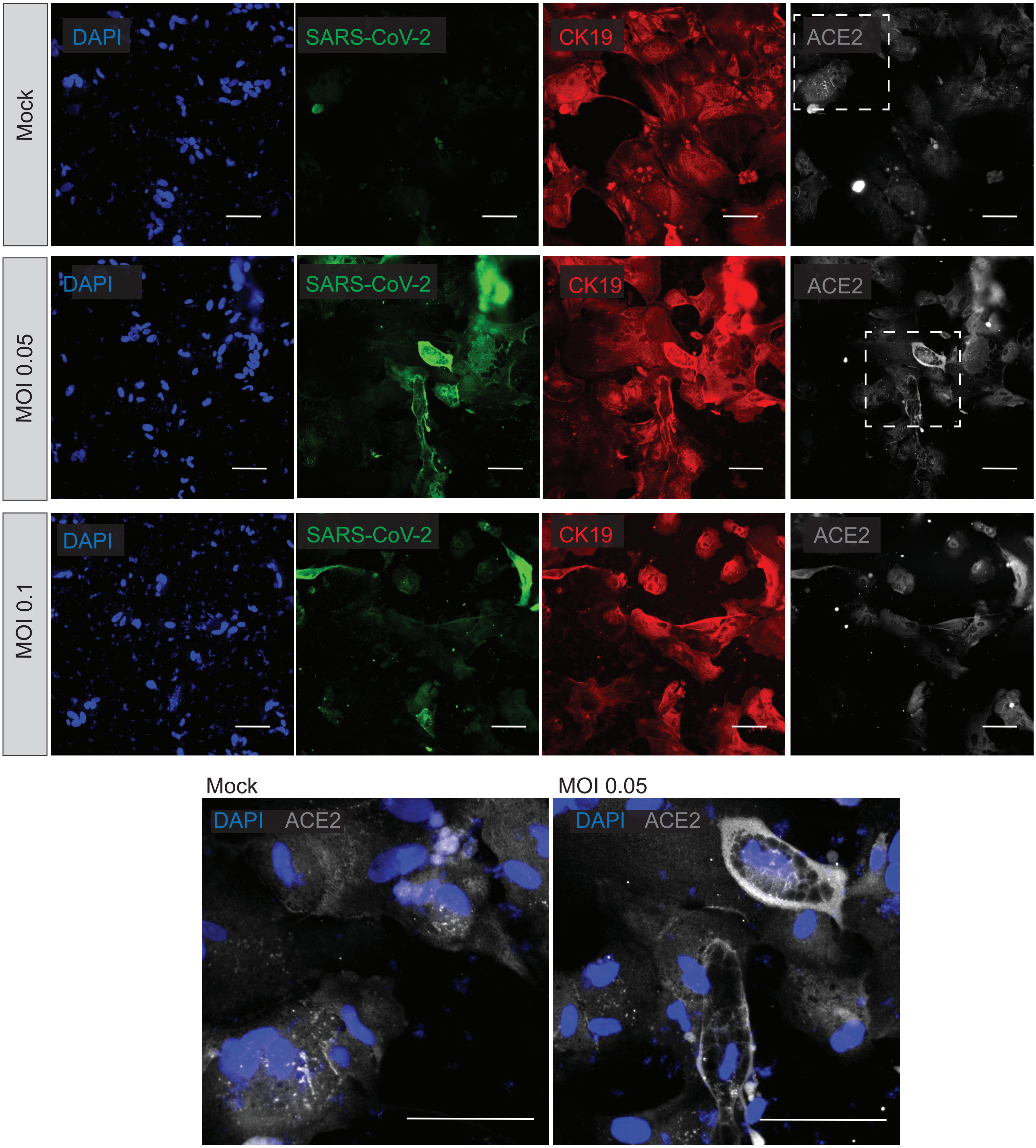

Supplementary Fig 2. iPan^{EXO} ductal cells express ACE2. Day 3 post infected iPan^{EXO} ductal cells exhibit ACE2 expression in SARS-CoV-2 infected and uninfected Mock control groups. The images below show the maginfied regions of interest of DAPI and ACE2 staining in the dotted line boxes shown in the main panel for Mock and MOI 0.05 groups. Scale bar represents 200 μm. ICC images shown here are representative results of 36 independent sites acquired. These results were pooled from 2 independent rounds of virus infections.

Supplementary Figure 3

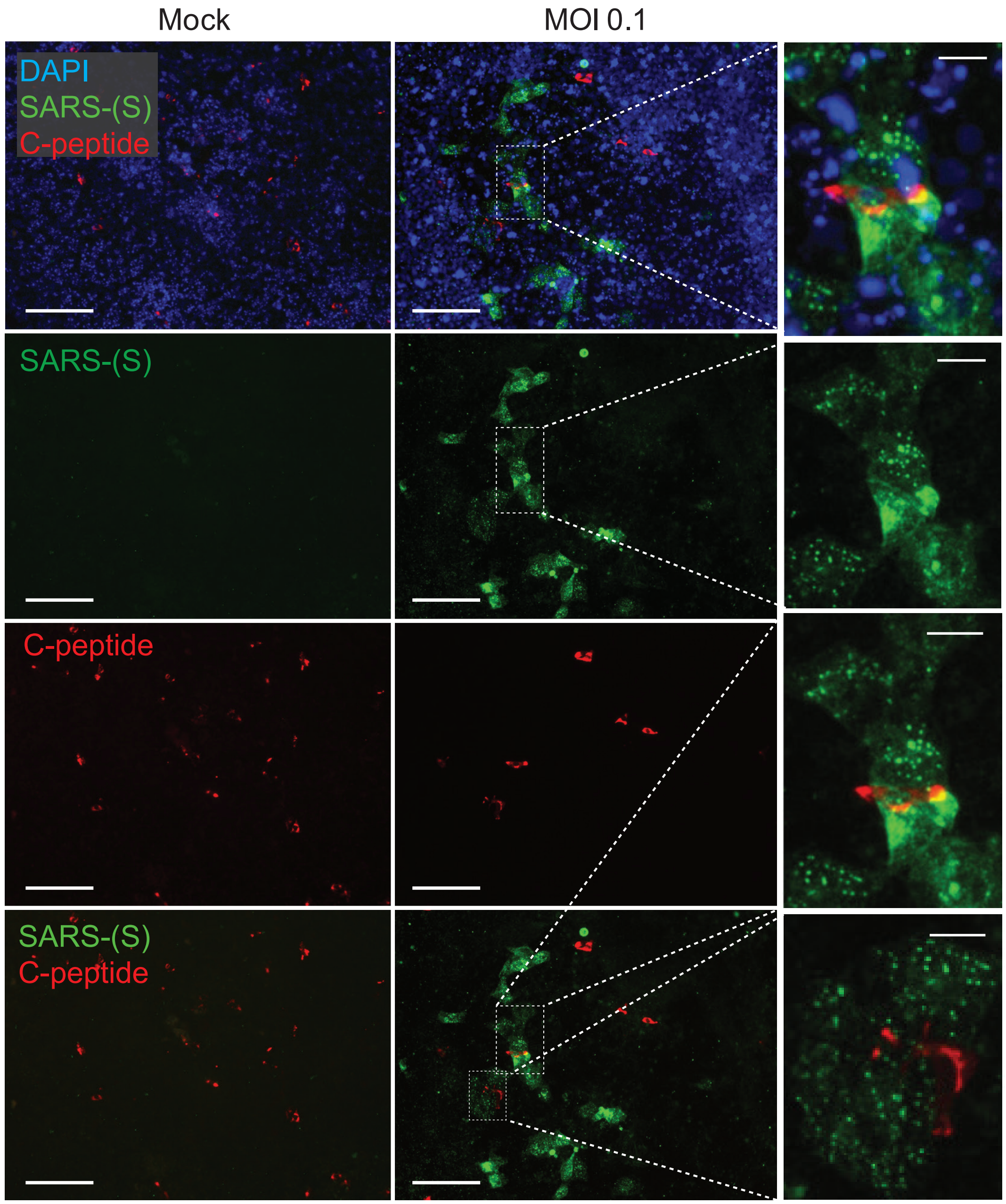

Supplementary Fig 3. C-peptide positive cells are infected by SARS-CoV-2. iPan^{EXO} acinar cultures contain small clusters of C-peptide protein expressing cells. A portion of these C-peptide positive cells were found to additionally express SARS-CoV-2-(S) on Day 3 (72 hours post-infection). Scale bar represents 130 μm, and 20 μm for zoomed panels adjacent to main images. ICC images shown here are representative results of 27 independent sites acquired. These results were pooled from 2 independent rounds of virus infections.

Supplementary Figure 4

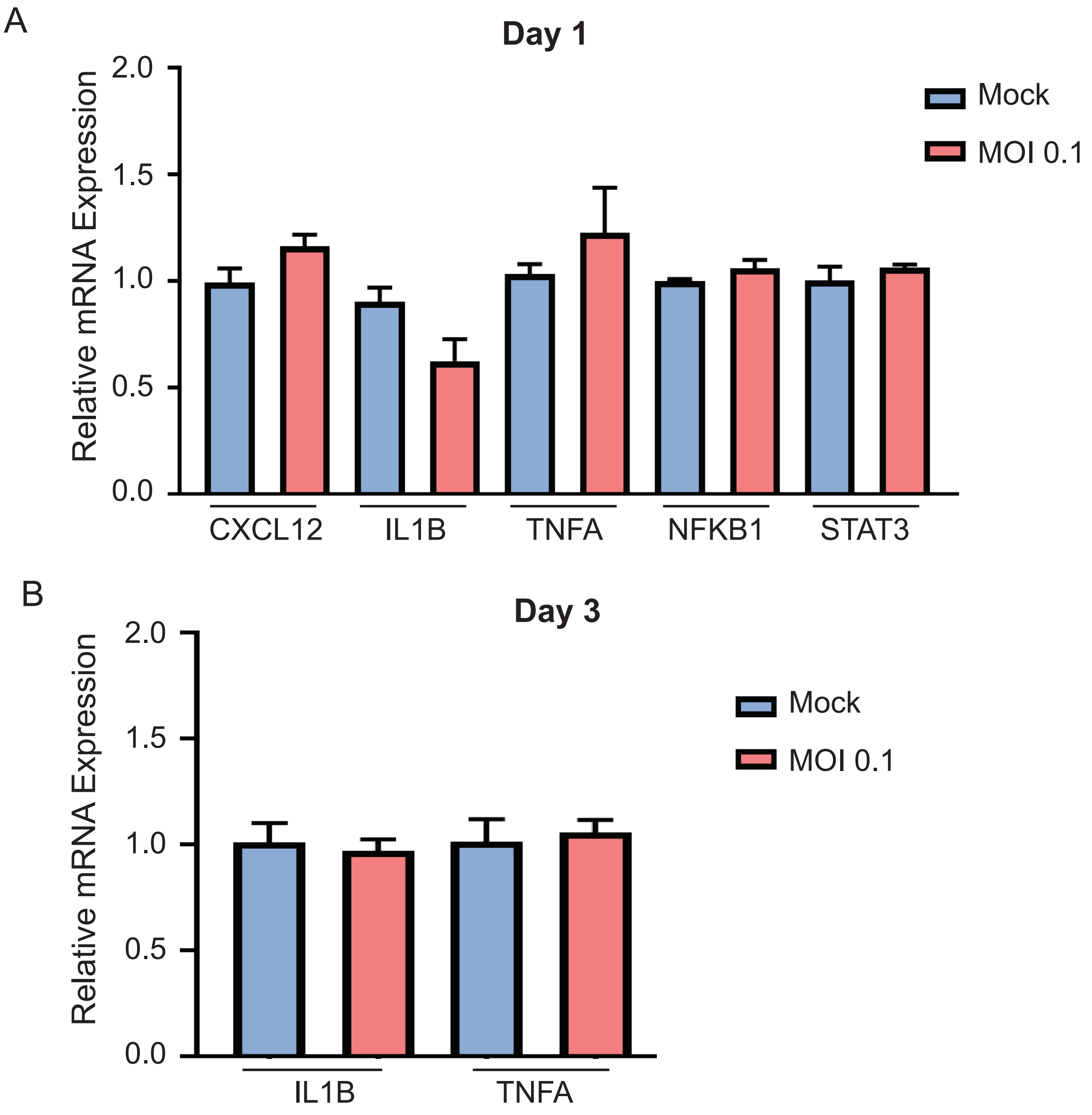

Supplementary Fig 4. Inflammatory markers were not altered by SARS-CoV-2 infection at Day 1 and as well as *IL1B* and *TNFA* at Day 3. A. RT-qPCR of infected and non-infected samples collected 24 hours (Day 1) after infection do not show significant change of mRNA expression of *CXCL12, IL1B, TNFA, NFKB1*, or *STAT3*. **B.** RT-qPCR of infected and non-infected samples collected 72 hours (Day 3) after infection do not show significant change of *IL1B* or *TNFA* mRNA expression. Data is shown as mean ± SEM with statistical significance determined by unpaired two-tailed t-test. RNA was extracted from aggregates of 16 biological replicates (using 96-well plate), and qPCR was run in 3 technical replicates per sample. These results were pooled from 2 independent rounds of virus infections.

Supplementary Figure 5

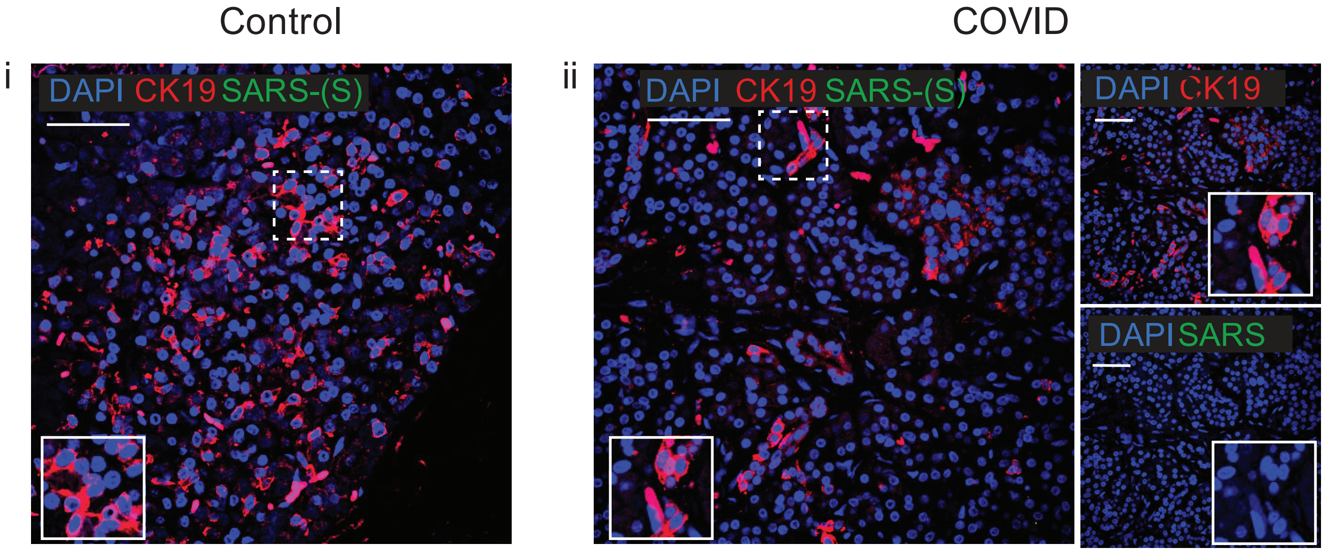

Supplementary Fig 5. Post-Mortem Human Pancreas Staining of pancreatic ductal marker Cytokeratin 19. Control and COVID-19 patients do not exhibit SARS-CoV-2 Spike S1 protein (green) in pancreatic ductal population, CK19 (red). Scale bar represents 50 µM. IHC was performed in 3 non-COVID control and 3 COVID patients. The results shown here are representative 3-10 independent sites acquired per patient.

Supplementary Figure 6

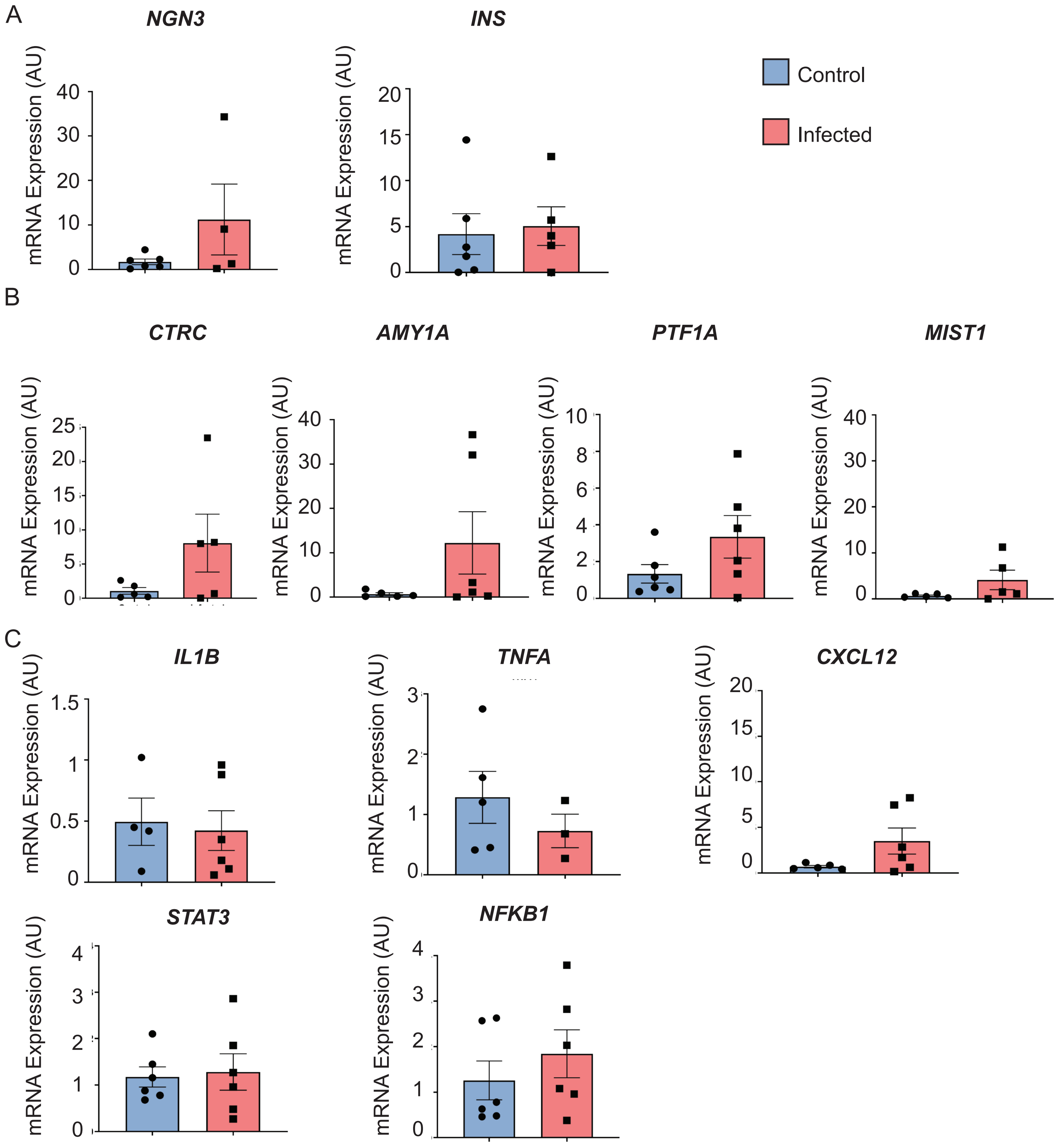

Supplementary Fig 6. mRNA expression of additional pancreatic and inflammatory markers from post-mortem COVID-19 and Control patients. Post-mortem pancreatic tissues from COVID-19 patients (n=6) and non-COVID controls (n=5) were probed for mRNA expression of **A.** endocrine markers *NGN3* and *INS*; **B.** acinar markers *CTRC, AMY1A*, *PTF1A* and *MIST1*; and **C.** inflammatory markers *IL1B*, *TNFA*, *CXCL12*, *NFKB1* and *STAT3.* Data is shown as mean ± SEM with statistical significance determined by unpaired two-tailed t-test. RNA was extracted from 5 non-COVID controls and 6 COVID patients. qPCR was run in 3 technical replicates per sample.

Supplementary Table 1. Formulation of base media for culture of iPSC-derived iPan^{EXO} Acinar and Ductal cells.

Phase I & II base medium		
**Component**	**Amount**	**Final concentration**
MCDB131 (FisherScientific)	98 ml	
Glutamax (FisherScientific)	1 ml	2 mM
Vitamin C (Sigma)	100 µl	250 µM
BSA (VWR)	500 mg	0.5%
$NaHCO_3$ (Sigma)	150 mg	1.5 g/L
Pen/Strep (Sigma)	1 ml	1%
**Phase III base medium**		
**Component**	**Amount**	**Final concentration**
DMEM (ThermoFisher)	98 ml	
Vitamin C	100 µl	250 µM
B27 supplement without Vitamin A (ThermoFisher)	100 µl	1%
Pen/Strep	1 ml	1%

Suppl. Table 2 List of primer sequences used for Real-Time qPCR.

Gene	Forward (5´-3´)	Reverse (5´-3´)
β-ACTIN	TGACAGGATGCAGAAGGAGA	CGCTCAGGAGGAGCAATG
GAPDH	CCACCTTTGACGCTGGG	CATACCAGGAAATGAGCTTGACA
RPL13	GGCTAAACAGGTACTGCTGGG	AGGAAAGCCAGGTACTTCAACTT
ACE2	TCCAGACTCCGATCATCAAGC	TGCTCATGGTGTTCAGAATTGT
TMPRSS2	TATGAGAACCACGGGTATCAGT	CGTTGTAATCCTCGGAGCATACT
SARS-CoV-2	GACCCCAAAATCAGCGAAAT	TCTGGTTACTGCCAGTTGAATCTG
NFKB1	TCCATATTTGGGAAGGCCTGA	TTGAAGGTATGGGCCATCTGT
CXCL12	GACAAGTGTGCATTGACCCG	TCTCACATCTTGAACCTCTTGT
IL1B	AGCCATGGCAGAAGTACCTG	CCTGGAAGGAGCACTTCATCT
TNFA	CCCCAGGGACCTCTCTCTAAT	GCTTGAGGGTTTGCTACAAC
STAT3	ACCATTGACCTGCCGATGTC	AAGGTGAGGGACTCAAACTGC
AMY2A	TGTAACAATGTTGGGGTTCGT	CACTCACAGCGTTACCACAC
CA2	TCAGGACAAAGCAGTGCTCAA	TGTCCATCAAGTGAACCCCAG
CFTR	TGACCTTCTGCCTCTTACCA	CACTATCACTGGCACTGTTGC
CTRC	CCGCCTCTGGACCAACG	TGAAGCCCCACCAGTCAATC
HNF1B	GCACCCCTATGAAGACCCAG	GGACTGTCTGGTTGAATTGTCG
INS	AGGCCATCAAGCAGATCACT	TTCCCCGCACACTAGGTAGA
KRT19	GTTCACCAGCCGGACTGAA	GCAGGTCAGTAACCTCGGAC
MIST1	TCCAAGATCGAGACGCTCAC	TGCTGGACATGGTCAGGATG
NGN3	CGGTAGAAAGGATGACGCCT	GGTCACTTCGTCTTCCGAGG
PTF1A	TCGGGGCACCCGGTC	AGAGAGAGTGTCCTGCTAGGG

Supplementary Table 3. Expression of Viral Transcripts in RNAseq data.

	Day 1 Samples						Day 3 Samples					
	Mock			Infected			Mock			Infected		
	M1	M2	M3	COV1	COV2	COV3	M4	M5	M6	COV4	COV5	COV6
id_SARS_COV_2 _MT246667_ 1_1_261	-	-	-	162	192	198	-	-	-	616	823	815
virus_E	-	-	-	22	26	32	-	-	-	160	177	165
virus_M	-	-	-	265	276	293	-	-	-	1,488	1,828	1,891
virus_N	-	1	-	2,008	2,105	2,384	-	-	-	10,183	11,917	13,240
virus_ORF10	-	-	-	84	72	65	-	-	-	422	485	434
virus_ORF3a	-	-	-	80	111	108	-	-	-	697	854	861
virus_ORF6	-	-	-	25	29	31	-	-	-	148	167	136
virus_ORF7a	-	-	-	173	168	189	-	-	-	990	1,199	1,276
virus_ORF8	-	-	-	193	209	251	-	-	-	1,244	1,531	1,479
virus_S	-	-	-	193	227	230	-	-	-	2,054	2,474	2,483
virus_orf1ab	-	-	-	325	336	333	-	-	-	2,333	2,837	3,176
**Virus Total**	-	**1**	-	**3,530**	**3,751**	**4,114**	-	-	-	**20,335**	**24,292**	**25,956**
**Total Mapped Reads**	**2.95 E7**	**2.84 E7**	**3.26 E7**	**3.04 E7**	**3.18 E7**	**3.34 E7**	**2.89 E7**	**3.13 E7**	**2.46 E7**	**2.51 E7**	**3.02 E7**	**3.28 E7**

Transcripts from the viral genome were detected within infected cell cultures. The count number of sequenced reads which mapped to the viral genome are listed above. M1, M2, M3, M4, M5, M6= indicate Mock conditions. COV1, COV2, COV3, COV4, COV5, COV6= indicate COVID-19 conditions.

Supplementary Table 4. Significant Differentially Expressed Genes Between SARS-CoV-2 Infected and Uninfected Cultures

Ensembl ID	Gene	Description	Log2FC	Adjusted p-value
ENSG00000283765	AC131160.1	presenilin associated rhomboid like	9.54	8.440E-12
ENSG00000257921	AC025165.3	novel protein	4.32	8.610E-06
ENSG00000177984	LCN15	lipocalin 15	1.83	7.730E-05
ENSG00000257390	AC023055.1	novel protein	1.77	6.450E-05
ENSG00000106483	SFRP4	secreted frizzled related protein 4	1.72	3.869E-04
ENSG00000145721	LIX1	limb and CNS expressed 1	1.53	3.350E-06
ENSG00000117318	ID3	inhibitor of DNA binding 3, HLH protein	1.46	1.537E-02
ENSG00000164107	HAND2	heart and neural crest derivatives expressed 2	1.44	3.270E-05
ENSG00000101230	ISM1	isthmin 1	1.35	4.765E-02
ENSG00000166482	MFAP4	microfibril associated protein 4	1.33	2.052E-02
ENSG00000115263	GCG	glucagon	1.28	3.950E-07
ENSG00000163359	COL6A3	collagen type VI alpha 3 chain	1.24	5.520E-11
ENSG00000124216	SNAI1	snail family transcriptional repressor 1	1.24	4.108E-02
ENSG00000130700	GATA5	GATA binding protein 5	1.22	1.219E-03
ENSG00000187634	SAMD11	sterile alpha motif domain containing 11	1.19	2.616E-03
ENSG00000099960	SLC7A4	solute carrier family 7 member 4	1.14	5.305E-03
ENSG00000287542	AC098582.1	HECT and RLD domain containing E3 ubiquitin protein ligase 3	1.14	1.097E-02
ENSG00000079112	CDH17	cadherin 17	1.13	2.100E-06
ENSG00000116132	PRRX1	paired related homeobox 1	1.13	1.578E-02
ENSG00000159251	ACTC1	actin alpha cardiac muscle 1	1.12	2.330E-10
ENSG00000090402	SI	sucrase-isomaltase	1.11	1.063E-02
ENSG00000106366	SERPINE1	serpin family E member 1	1.11	2.575E-02
ENSG00000169903	TM4SF4	transmembrane 4 L six family member 4	1.1	2.841E-04
ENSG00000154188	ANGPT1	angiopoietin 1	1.1	8.086E-03
ENSG00000240583	AQP1	aquaporin 1 (Colton blood group)	1.1	3.698E-02
ENSG00000254647	INS	insulin	1.09	7.400E-08
ENSG00000179921	GPBAR1	G protein-coupled bile acid receptor 1	1.08	4.645E-02
ENSG00000107731	UNC5B	unc-5 netrin receptor B	1.07	2.616E-03
ENSG00000136698	CFC1	cripto, FRL-1, cryptic family 1	1.05	2.061E-03
ENSG00000115738	ID2	inhibitor of DNA binding 2	1.02	1.590E-21
ENSG00000151834	GABRA2	gamma-aminobutyric acid type A receptor subunit alpha2	1.02	7.830E-05
ENSG00000120149	MSX2	msh homeobox 2	1	3.060E-06
ENSG00000112175	BMP5	bone morphogenetic protein 5	1	2.690E-05
ENSG00000157017	GHRL	ghrelin and obestatin prepropeptide	0.97	5.510E-21
ENSG00000054598	FOXC1	forkhead box C1	0.96	1.959E-02

ENSG00000135111	TBX3	T-box transcription factor 3	0.95	1.458E-04
ENSG00000213759	UGT2B11	UDP glucuronosyltransferase family 2 member B11	0.94	8.160E-06
ENSG00000205038	PKHD1L1	PKHD1 like 1	0.91	2.843E-02
ENSG00000172780	RAB43	RAB43, member RAS oncogene family	0.87	6.920E-06
ENSG00000248329	APELA	apelin receptor early endogenous ligand	0.87	2.530E-05
ENSG00000183145	RIPPLY3	ripply transcriptional repressor 3	0.87	1.044E-02
ENSG00000139515	PDX1	pancreatic and duodenal homeobox 1	0.84	1.632E-03
ENSG00000165092	ALDH1A1	aldehyde dehydrogenase 1 family member A1	0.84	4.214E-02
ENSG00000119946	CNNM1	cyclin and CBS domain divalent metal cation transport mediator 1	0.83	1.655E-02
ENSG00000124875	CXCL6	C-X-C motif chemokine ligand 6	0.82	2.915E-02
ENSG00000148344	PTGES	prostaglandin E synthase	0.8	5.740E-09
ENSG00000159173	TNNI1	troponin I1, slow skeletal type	0.8	4.764E-02
ENSG00000163453	IGFBP7	insulin like growth factor binding protein 7	0.78	5.860E-10
ENSG00000284395	PERCC1	proline and glutamate rich with coiled coil 1	0.75	3.322E-02
ENSG00000172058	SERF1A	small EDRK-rich factor 1A	0.74	5.526E-04
ENSG00000122691	TWIST1	twist family bHLH transcription factor 1	0.74	1.814E-02
ENSG00000168542	COL3A1	collagen type III alpha 1 chain	0.73	1.670E-08
ENSG00000130829	DUSP9	dual specificity phosphatase 9	0.73	2.395E-02
ENSG00000107562	CXCL12	C-X-C motif chemokine ligand 12	0.72	2.944E-02
ENSG00000095739	BAMBI	BMP and activin membrane bound inhibitor	0.71	8.908E-04
ENSG00000115363	EVA1A	eva-1 homolog A, regulator of programmed cell death	0.7	1.822E-02
ENSG00000154162	CDH12	cadherin 12	0.69	1.607E-02
ENSG00000136842	TMOD1	tropomodulin 1	0.68	3.036E-02
ENSG00000101665	SMAD7	SMAD family member 7	0.67	9.751E-03
ENSG00000103196	CRISPLD2	cysteine rich secretory protein LCCL domain containing 2	0.65	1.190E-05
ENSG00000135744	AGT	angiotensinogen	0.65	3.273E-03
ENSG00000129521	EGLN3	egl-9 family hypoxia inducible factor 3	0.64	7.398E-03
ENSG00000130822	PNCK	pregnancy up-regulated nonubiquitous CaM kinase	0.64	1.737E-02
ENSG00000181234	TMEM132C	transmembrane protein 132C	0.64	2.145E-02
ENSG00000280987	MATR3	matrin 3	0.64	3.980E-02
ENSG00000173826	KCNH6	potassium voltage-gated channel subfamily H member 6	0.64	4.975E-02
ENSG00000135406	PRPH	peripherin	0.63	2.640E-02

ENSG00000185559	DLK1	delta like non-canonical Notch ligand 1	0.61	1.190E-05
ENSG00000164237	CMBL	carboxymethylenebutenolidase homolog	0.61	8.304E-03
ENSG00000124762	CDKN1A	cyclin dependent kinase inhibitor 1A	0.6	4.200E-06
ENSG00000079689	SCGN	secretagogin, EF-hand calcium binding protein	0.6	2.048E-02
ENSG00000187720	THSD4	thrombospondin type 1 domain containing 4	0.6	4.379E-02
ENSG00000128606	LRRC17	leucine rich repeat containing 17	0.6	4.911E-02
ENSG00000135480	KRT7	keratin 7	0.59	3.957E-03
ENSG00000134013	LOXL2	lysyl oxidase like 2	0.59	2.567E-02
ENSG00000115828	QPCT	glutaminyl-peptide cyclotransferase	0.59	4.308E-02
ENSG00000265972	TXNIP	thioredoxin interacting protein	0.58	7.500E-11
ENSG00000100604	CHGA	chromogranin A	0.58	8.629E-03
ENSG00000122547	EEPD1	endonuclease/exonuclease/phosphatase family domain containing 1	0.58	3.800E-02
ENSG00000146674	IGFBP3	insulin like growth factor binding protein 3	0.56	1.483E-02
ENSG00000090013	BLVRB	biliverdin reductase B	0.56	2.145E-02
ENSG00000175426	PCSK1	proprotein convertase subtilisin/kexin type 1	0.56	4.549E-02
ENSG00000001626	CFTR	CF transmembrane conductance regulator	0.55	1.403E-02
ENSG00000103260	METRN	meteorin, glial cell differentiation regulator	0.55	3.572E-02
ENSG00000166165	CKB	creatine kinase B	0.53	1.560E-10
ENSG00000130429	ARPC1B	actin related protein 2/3 complex subunit 1B	0.53	6.667E-04
ENSG00000108821	COL1A1	collagen type I alpha 1 chain	0.53	2.778E-02
ENSG00000115221	ITGB6	integrin subunit beta 6	0.52	3.816E-04
ENSG00000187840	EIF4EBP1	eukaryotic translation initiation factor 4E binding protein 1	0.52	3.645E-03
ENSG00000105143	SLC1A6	solute carrier family 1 member 6	0.52	1.011E-02
ENSG00000140450	ARRDC4	arrestin domain containing 4	0.51	3.950E-07
ENSG00000154734	ADAMTS1	ADAM metallopeptidase with thrombospondin type 1 motif 1	0.51	2.841E-04
ENSG00000178057	NDUFAF3	NADH:ubiquinone oxidoreductase complex assembly factor 3	0.51	5.676E-03
ENSG00000133027	PEMT	phosphatidylethanolamine N-methyltransferase	0.51	8.706E-03
ENSG00000065057	NTHL1	nth like DNA glycosylase 1	0.51	2.785E-02
ENSG00000169220	RGS14	regulator of G protein signaling 14	0.51	4.238E-02
ENSG00000049130	KITLG	KIT ligand	0.5	9.760E-05
ENSG00000123358	NR4A1	nuclear receptor subfamily 4 group A member 1	0.5	4.394E-03
ENSG00000165066	NKX6-3	NK6 homeobox 3	0.5	7.698E-03

ENSG00000110104	CCDC86	coiled-coil domain containing 86	0.5	1.454E-02
ENSG00000183048	SLC25A10	solute carrier family 25 member 10	0.5	3.839E-02
ENSG00000175793	SFN	stratifin	0.5	4.660E-02
ENSG00000157005	SST	somatostatin	0.49	2.263E-02
ENSG00000063241	ISOC2	isochorismatase domain containing 2	0.49	2.372E-02
ENSG00000126218	F10	coagulation factor X	0.49	4.306E-02
ENSG00000112576	CCND3	cyclin D3	0.48	1.097E-02
ENSG00000165272	AQP3	aquaporin 3 (Gill blood group)	0.48	1.233E-02
ENSG00000112276	BVES	blood vessel epicardial substance	0.48	3.575E-02
ENSG00000171159	C9orf16	chromosome 9 open reading frame 16	0.47	1.302E-02
ENSG00000134258	VTCN1	V-set domain containing T cell activation inhibitor 1	0.46	9.290E-07
ENSG00000205544	TMEM256	transmembrane protein 256	0.46	4.130E-06
ENSG00000162878	PKDCC	protein kinase domain containing, cytoplasmic	0.46	1.600E-05
ENSG00000151090	THRB	thyroid hormone receptor beta	0.46	4.802E-03
ENSG00000120708	TGFBI	transforming growth factor beta induced	0.46	5.522E-03
ENSG00000152082	MZT2B	mitotic spindle organizing protein 2B	0.46	1.195E-02
ENSG00000183010	PYCR1	pyrroline-5-carboxylate reductase 1	0.46	2.313E-02
ENSG00000196975	ANXA4	annexin A4	0.45	2.776E-03
ENSG00000122863	CHST3	carbohydrate sulfotransferase 3	0.45	1.328E-02
ENSG00000127561	SYNGR3	synaptogyrin 3	0.45	4.771E-02
ENSG00000161958	FGF11	fibroblast growth factor 11	0.44	1.728E-04
ENSG00000087245	MMP2	matrix metallopeptidase 2	0.44	1.674E-02
ENSG00000141526	SLC16A3	solute carrier family 16 member 3	0.44	4.737E-02
ENSG00000151726	ACSL1	acyl-CoA synthetase long chain family member 1	0.43	6.520E-08
ENSG00000083845	RPS5	ribosomal protein S5	0.43	3.050E-07
ENSG00000141448	GATA6	GATA binding protein 6	0.43	1.020E-03
ENSG00000168000	BSCL2	BSCL2 lipid droplet biogenesis associated, seipin	0.43	4.453E-03
ENSG00000109861	CTSC	cathepsin C	0.42	1.632E-03
ENSG00000181026	AEN	apoptosis enhancing nuclease	0.42	2.537E-03
ENSG00000034152	MAP2K3	mitogen-activated protein kinase kinase 3	0.42	6.236E-03
ENSG00000101220	C20orf27	chromosome 20 open reading frame 27	0.42	1.086E-02
ENSG00000125170	DOK4	docking protein 4	0.42	1.737E-02
ENSG00000172731	LRRC20	leucine rich repeat containing 20	0.41	1.972E-02
ENSG00000120885	CLU	clusterin	0.41	4.179E-02
ENSG00000124145	SDC4	syndecan 4	0.4	1.821E-04
ENSG00000180914	OXTR	oxytocin receptor	0.4	3.074E-04
ENSG00000181788	SIAH2	siah E3 ubiquitin protein ligase 2	0.4	1.172E-02
ENSG00000117691	NENF	neudesin neurotrophic factor	0.4	1.271E-02

ENSG00000155254	MARVELD1	MARVEL domain containing 1	0.4	1.420E-02
ENSG00000115884	SDC1	syndecan 1	0.39	4.190E-05
ENSG00000131469	RPL27	ribosomal protein L27	0.39	1.458E-04
ENSG00000267855	NDUFA7	NADH:ubiquinone oxidoreductase subunit A7	0.39	7.333E-03
ENSG00000105372	RPS19	ribosomal protein S19	0.38	1.979E-04
ENSG00000074370	ATP2A3	ATPase sarcoplasmic/endoplasmic reticulum Ca2+ transporting 3	0.38	7.087E-04
ENSG00000176946	THAP4	THAP domain containing 4	0.38	1.777E-03
ENSG00000145685	LHFPL2	LHFPL tetraspan subfamily member 2	0.38	1.985E-02
ENSG00000140937	CDH11	cadherin 11	0.37	4.134E-03
ENSG00000110108	TMEM109	transmembrane protein 109	0.37	4.823E-03
ENSG00000165716	DIPK1B	divergent protein kinase domain 1B	0.37	1.097E-02
ENSG00000011260	UTP18	UTP18 small subunit processome component	0.37	1.362E-02
ENSG00000142327	RNPEPL1	arginyl aminopeptidase like 1	0.37	3.783E-02
ENSG00000140564	FURIN	furin, paired basic amino acid cleaving enzyme	0.37	4.496E-02
ENSG00000164587	RPS14	ribosomal protein S14	0.36	4.090E-06
ENSG00000054148	PHPT1	phosphohistidine phosphatase 1	0.36	1.455E-04
ENSG00000189143	CLDN4	claudin 4	0.36	2.943E-04
ENSG00000090776	EFNB1	ephrin B1	0.36	5.803E-04
ENSG00000088756	ARHGAP28	Rho GTPase activating protein 28	0.36	1.097E-02
ENSG00000130332	LSM7	LSM7 homolog, U6 small nuclear RNA and mRNA degradation associated	0.36	1.134E-02
ENSG00000183172	SMDT1	single-pass membrane protein with aspartate rich tail 1	0.36	2.001E-02
ENSG00000095906	NUBP2	nucleotide binding protein 2	0.36	2.111E-02
ENSG00000166595	CIAO2B	cytosolic iron-sulfur assembly component 2B	0.36	2.700E-02
ENSG00000275993	SIK1B	salt inducible kinase 1B (putative)	0.36	4.496E-02
ENSG00000105640	RPL18A	ribosomal protein L18a	0.35	1.930E-05
ENSG00000103257	SLC7A5	solute carrier family 7 member 5	0.35	3.070E-05
ENSG00000164638	SLC29A4	solute carrier family 29 member 4	0.35	4.031E-03
ENSG00000148834	GSTO1	glutathione S-transferase omega 1	0.35	1.619E-02
ENSG00000177556	ATOX1	antioxidant 1 copper chaperone	0.35	2.472E-02
ENSG00000131043	AAR2	AAR2 splicing factor	0.35	3.839E-02
ENSG00000077238	IL4R	interleukin 4 receptor	0.35	4.911E-02
ENSG00000240972	MIF	macrophage migration inhibitory factor	0.34	1.720E-03
ENSG00000138449	SLC40A1	solute carrier family 40 member 1	0.34	2.488E-03
ENSG00000233927	RPS28	ribosomal protein S28	0.34	3.645E-03
ENSG00000165672	PRDX3	peroxiredoxin 3	0.34	1.030E-02
ENSG00000112559	MDFI	MyoD family inhibitor	0.34	1.441E-02
ENSG00000006282	SPATA20	spermatogenesis associated 20	0.34	2.012E-02

ENSG00000214063	TSPAN4	tetraspanin 4	0.34	3.790E-02
ENSG00000123179	EBPL	EBP like	0.34	4.310E-02
ENSG00000121900	TMEM54	transmembrane protein 54	0.34	4.423E-02
ENSG00000109475	RPL34	ribosomal protein L34	0.33	5.175E-04
ENSG00000068001	HYAL2	hyaluronidase 2	0.33	6.018E-04
ENSG00000198931	APRT	adenine phosphoribosyltransferase	0.33	1.097E-02
ENSG00000221983	UBA52	ubiquitin A-52 residue ribosomal protein fusion product 1	0.33	1.221E-02
ENSG00000109736	MFSD10	major facilitator superfamily domain containing 10	0.33	1.354E-02
ENSG00000185664	PMEL	premelanosome protein	0.33	1.483E-02
ENSG00000120256	LRP11	LDL receptor related protein 11	0.33	2.376E-02
ENSG00000105193	RPS16	ribosomal protein S16	0.33	2.966E-02
ENSG00000171604	CXXC5	CXXC finger protein 5	0.33	3.389E-02
ENSG00000123992	DNPEP	aspartyl aminopeptidase	0.33	3.786E-02
ENSG00000152078	TLCD4	TLC domain containing 4	0.33	3.839E-02
ENSG00000063177	RPL18	ribosomal protein L18	0.33	4.310E-02
ENSG00000167695	TLCD3A	TLC domain containing 3A	0.33	4.516E-02
ENSG00000239264	TXNDC5	thioredoxin domain containing 5	0.32	5.180E-05
ENSG00000178202	POGLUT3	protein O-glucosyltransferase 3	0.32	3.686E-03
ENSG00000147403	RPL10	ribosomal protein L10	0.32	4.353E-03
ENSG00000204392	LSM2	LSM2 homolog, U6 small nuclear RNA and mRNA degradation associated	0.32	6.337E-03
ENSG00000130635	COL5A1	collagen type V alpha 1 chain	0.32	1.029E-02
ENSG00000115268	RPS15	ribosomal protein S15	0.32	1.043E-02
ENSG00000125995	ROMO1	reactive oxygen species modulator 1	0.32	2.587E-02
ENSG00000137801	THBS1	thrombospondin 1	0.32	4.529E-02
ENSG00000105677	TMEM147	transmembrane protein 147	0.32	4.549E-02
ENSG00000133243	BTBD2	BTB domain containing 2	0.32	4.600E-02
ENSG00000205542	TMSB4X	thymosin beta 4 X-linked	0.31	1.260E-05
ENSG00000188986	NELFB	negative elongation factor complex member B	0.31	2.907E-03
ENSG00000166886	NAB2	NGFI-A binding protein 2	0.31	6.148E-03
ENSG00000143811	PYCR2	pyrroline-5-carboxylate reductase 2	0.31	1.097E-02
ENSG00000106153	CHCHD2	coiled-coil-helix-coiled-coil-helix domain containing 2	0.31	1.497E-02
ENSG00000071082	RPL31	ribosomal protein L31	0.31	1.498E-02
ENSG00000102265	TIMP1	TIMP metallopeptidase inhibitor 1	0.31	4.109E-02
ENSG00000198840	MT-ND3	mitochondrially encoded NADH:ubiquinone oxidoreductase core subunit 3	0.31	4.472E-02
ENSG00000198242	RPL23A	ribosomal protein L23a	0.3	3.005E-04
ENSG00000116857	TMEM9	transmembrane protein 9	0.3	7.041E-04
ENSG00000165283	STOML2	stomatin like 2	0.3	2.877E-03
ENSG00000100814	CCNB1IP1	cyclin B1 interacting protein 1	0.3	3.112E-03
ENSG00000198918	RPL39	ribosomal protein L39	0.3	3.645E-03

ENSG00000143543	JTB	jumping translocation breakpoint	0.3	8.694E-03
ENSG00000204388	HSPA1B	heat shock protein family A (Hsp70) member 1B	0.3	2.133E-02
ENSG00000149806	FAU	FAU ubiquitin like and ribosomal protein S30 fusion	0.3	4.050E-02
ENSG00000117419	ERI3	ERI1 exoribonuclease family member 3	0.3	4.975E-02
ENSG00000127184	COX7C	cytochrome c oxidase subunit 7C	0.29	1.539E-04
ENSG00000101444	AHCY	adenosylhomocysteinase	0.29	3.576E-04
ENSG00000132507	EIF5A	eukaryotic translation initiation factor 5A	0.29	3.643E-04
ENSG00000149948	HMGA2	high mobility group AT-hook 2	0.29	4.938E-04
ENSG00000164292	RHOBTB3	Rho related BTB domain containing 3	0.29	3.871E-03
ENSG00000136240	KDELR2	KDEL endoplasmic reticulum protein retention receptor 2	0.29	6.834E-03
ENSG00000231500	RPS18	ribosomal protein S18	0.29	8.305E-03
ENSG00000084207	GSTP1	glutathione S-transferase pi 1	0.29	1.100E-02
ENSG00000177830	CHID1	chitinase domain containing 1	0.29	1.362E-02
ENSG00000183098	GPC6	glypican 6	0.29	1.407E-02
ENSG00000171858	RPS21	ribosomal protein S21	0.29	1.656E-02
ENSG00000170892	TSEN34	tRNA splicing endonuclease subunit 34	0.29	2.785E-02
ENSG00000162244	RPL29	ribosomal protein L29	0.29	2.902E-02
ENSG00000172809	RPL38	ribosomal protein L38	0.29	3.449E-02
ENSG00000198951	NAGA	alpha-N-acetylgalactosaminidase	0.29	3.843E-02
ENSG00000167543	TP53I13	tumor protein p53 inducible protein 13	0.29	4.549E-02
ENSG00000176101	SSNA1	SS nuclear autoantigen 1	0.29	4.660E-02
ENSG00000198763	MT-ND2	mitochondrially encoded NADH:ubiquinone oxidoreductase core subunit 2	0.28	4.149E-03
ENSG00000184840	TMED9	transmembrane p24 trafficking protein 9	0.28	6.480E-03
ENSG00000117448	AKR1A1	aldo-keto reductase family 1 member A1	0.28	8.696E-03
ENSG00000172354	GNB2	G protein subunit beta 2	0.28	9.782E-03
ENSG00000170889	RPS9	ribosomal protein S9	0.28	1.254E-02
ENSG00000099797	TECR	trans-2,3-enoyl-CoA reductase	0.28	1.607E-02
ENSG00000134291	TMEM106C	transmembrane protein 106C	0.28	2.109E-02
ENSG00000179091	CYC1	cytochrome c1	0.28	3.246E-02
ENSG00000168003	SLC3A2	solute carrier family 3 member 2	0.28	3.914E-02
ENSG00000126768	TIMM17B	translocase of inner mitochondrial membrane 17B	0.28	4.011E-02
ENSG00000164692	COL1A2	collagen type I alpha 2 chain	0.28	4.459E-02
ENSG00000137288	UQCC2	ubiquinol-cytochrome c reductase complex assembly factor 2	0.28	4.879E-02

ENSG00000100316	RPL3	ribosomal protein L3	0.27	5.324E-04
ENSG00000143761	ARF1	ADP ribosylation factor 1	0.27	1.410E-02
ENSG00000167291	TBC1D16	TBC1 domain family member 16	0.27	2.108E-02
ENSG00000101182	PSMA7	proteasome 20S subunit alpha 7	0.27	3.337E-02
ENSG00000171551	ECEL1	endothelin converting enzyme like 1	0.27	4.448E-02
ENSG00000247596	TWF2	twinfilin actin binding protein 2	0.27	4.517E-02
ENSG00000107438	PDLIM1	PDZ and LIM domain 1	0.26	4.823E-03
ENSG00000175390	EIF3F	eukaryotic translation initiation factor 3 subunit F	0.26	6.513E-03
ENSG00000013306	SLC25A39	solute carrier family 25 member 39	0.26	1.254E-02
ENSG00000125166	GOT2	glutamic-oxaloacetic transaminase 2	0.26	1.853E-02
ENSG00000243678	NME2	NME/NM23 nucleoside diphosphate kinase 2	0.26	1.862E-02
ENSG00000172992	DCAKD	dephospho-CoA kinase domain containing	0.26	2.450E-02
ENSG00000204628	RACK1	receptor for activated C kinase 1	0.26	2.533E-02
ENSG00000124614	RPS10	ribosomal protein S10	0.26	4.052E-02
ENSG00000198755	RPL10A	ribosomal protein L10a	0.25	2.062E-03
ENSG00000075618	FSCN1	fascin actin-bundling protein 1	0.25	8.756E-03
ENSG00000213741	RPS29	ribosomal protein S29	0.25	1.362E-02
ENSG00000110651	CD81	CD81 molecule	0.25	2.376E-02
ENSG00000112514	CUTA	cutA divalent cation tolerance homolog	0.25	2.657E-02
ENSG00000182899	RPL35A	ribosomal protein L35a	0.25	2.902E-02
ENSG00000167283	ATP5MG	ATP synthase membrane subunit g	0.25	3.823E-02
ENSG00000126267	COX6B1	cytochrome c oxidase subunit 6B1	0.25	4.004E-02
ENSG00000140931	CMTM3	CKLF like MARVEL transmembrane domain containing 3	0.25	4.599E-02
ENSG00000116685	KIAA2013	KIAA2013	0.25	4.737E-02
ENSG00000084623	EIF3I	eukaryotic translation initiation factor 3 subunit I	0.24	2.575E-02
ENSG00000049239	H6PD	hexose-6-phosphate dehydrogenase/glucose 1-dehydrogenase	0.24	2.727E-02
ENSG00000149923	PPP4C	protein phosphatase 4 catalytic subunit	0.24	3.574E-02
ENSG00000169230	PRELID1	PRELI domain containing 1	0.24	3.644E-02
ENSG00000104635	SLC39A14	solute carrier family 39 member 14	0.24	3.812E-02
ENSG00000110987	BCL7A	BAF chromatin remodeling complex subunit BCL7A	0.24	3.839E-02
ENSG00000160691	SHC1	SHC adaptor protein 1	0.24	4.341E-02
ENSG00000125817	CENPB	centromere protein B	0.24	4.455E-02
ENSG00000131165	CHMP1A	charged multivesicular body protein 1A	0.24	4.459E-02
ENSG00000168028	RPSA	ribosomal protein SA	0.23	8.189E-04
ENSG00000089157	RPLP0	ribosomal protein lateral stalk subunit P0	0.23	9.471E-04

ENSG00000075624	ACTB	actin beta	0.23	1.078E-03
ENSG00000117592	PRDX6	peroxiredoxin 6	0.23	6.148E-03
ENSG00000137409	MTCH1	mitochondrial carrier 1	0.23	1.674E-02
ENSG00000155380	SLC16A1	solute carrier family 16 member 1	0.23	1.674E-02
ENSG00000124172	ATP5F1E	ATP synthase F1 subunit epsilon	0.23	1.845E-02
ENSG00000125835	SNRPB	small nuclear ribonucleoprotein polypeptides B and B1	0.23	2.850E-02
ENSG00000130726	TRIM28	tripartite motif containing 28	0.23	3.356E-02
ENSG00000100138	SNU13	small nuclear ribonucleoprotein 13	0.23	4.333E-02
ENSG00000130638	ATXN10	ataxin 10	0.22	2.450E-02
ENSG00000104738	MCM4	minichromosome maintenance complex component 4	0.22	2.510E-02
ENSG00000143418	CERS2	ceramide synthase 2	0.22	3.839E-02
ENSG00000197324	LRP10	LDL receptor related protein 10	0.22	4.023E-02
ENSG00000184009	ACTG1	actin gamma 1	0.22	4.139E-02
ENSG00000100129	EIF3L	eukaryotic translation initiation factor 3 subunit L	0.21	9.743E-03
ENSG00000173726	TOMM20	translocase of outer mitochondrial membrane 20	0.21	2.403E-02
ENSG00000110700	RPS13	ribosomal protein S13	0.21	3.511E-02
ENSG00000073111	MCM2	minichromosome maintenance complex component 2	0.21	3.773E-02
ENSG00000105202	FBL	fibrillarin	0.21	4.496E-02
ENSG00000168268	NT5DC2	5'-nucleotidase domain containing 2	0.21	4.597E-02
ENSG00000139644	TMBIM6	transmembrane BAX inhibitor motif containing 6	0.2	1.370E-02
ENSG00000108298	RPL19	ribosomal protein L19	0.2	3.836E-02
ENSG00000184117	NIPSNAP1	nipsnap homolog 1	0.19	2.275E-02
ENSG00000115484	CCT4	chaperonin containing TCP1 subunit 4	0.19	4.548E-02
ENSG00000075415	SLC25A3	solute carrier family 25 member 3	0.19	4.764E-02
ENSG00000112306	RPS12	ribosomal protein S12	0.18	4.307E-02
ENSG00000198938	MT-CO3	mitochondrially encoded cytochrome c oxidase III	0.16	4.939E-02
ENSG00000277277	FAM243B	family with sequence similarity 243 member B	-4.64	9.782E-03
ENSG00000272741	AC069257.4	novel protein	-3.89	2.523E-02
ENSG00000285258	ATXN7	NA	-2.84	1.410E-02
ENSG00000187642	PERM1	PPARGC1 and ESRR induced regulator, muscle 1	-2.83	2.327E-02
ENSG00000185040	SPDYE16	speedy/RINGO cell cycle regulator family member E16	-2.81	2.893E-02
ENSG00000261732	AL031708.1	novel protein	-2.27	3.960E-07
ENSG00000226763	SRRM5	serine/arginine repetitive matrix 5	-2.2	3.183E-02

ENSG00000249773	AC092647.5	novel zinc finger protein 713 (ZNF713) and mitochondrial ribosomal protein S17 (MRPS17) protein	-2.08	2.882E-04
ENSG00000205238	SPDYE2	speedy/RINGO cell cycle regulator family member E2	-2.04	6.480E-03
ENSG00000127364	TAS2R4	taste 2 receptor member 4	-1.97	2.133E-04
ENSG00000215018	COL28A1	collagen type XXVIII alpha 1 chain	-1.94	9.782E-03
ENSG00000272916	AC022400.7	novel transcript	-1.89	8.340E-06
ENSG00000089692	LAG3	lymphocyte activating 3	-1.83	3.585E-02
ENSG00000100078	PLA2G3	phospholipase A2 group III	-1.7	4.423E-02
ENSG00000170092	SPDYE5	speedy/RINGO cell cycle regulator family member E5	-1.69	7.398E-03
ENSG00000285130	AL358113.1	novel protein	-1.65	2.583E-02
ENSG00000186399	GOLGA8R	golgin A8 family member R	-1.42	4.496E-02
ENSG00000170160	CCDC144A	coiled-coil domain containing 144A	-1.37	6.796E-03
ENSG00000286053	ASDURF	ASNSD1 upstream reading frame	-1.37	3.350E-03
ENSG00000172005	MAL	mal, T cell differentiation protein	-1.36	3.356E-02
ENSG00000240053	LY6G5B	lymphocyte antigen 6 family member G5B	-1.34	9.330E-06
ENSG00000164707	SLC13A4	solute carrier family 13 member 4	-1.27	5.084E-03
ENSG00000164796	CSMD3	CUB and Sushi multiple domains 3	-1.26	5.433E-03
ENSG00000145113	MUC4	mucin 4, cell surface associated	-1.21	1.675E-03
ENSG00000242028	HYPK	huntingtin interacting protein K	-1.17	1.250E-05
ENSG00000204889	KRT40	keratin 40	-1.16	1.461E-02
ENSG00000170370	EMX2	empty spiracles homeobox 2	-1.14	6.610E-05
ENSG00000215252	GOLGA8B	golgin A8 family member B	-1.14	1.780E-06
ENSG00000166762	CATSPER2	cation channel sperm associated 2	-1.13	2.065E-04
ENSG00000135976	ANKRD36	ankyrin repeat domain 36	-1.12	1.410E-13
ENSG00000166432	ZMAT1	zinc finger matrin-type 1	-1.11	4.970E-05
ENSG00000274272	AC069281.2	novel transcript	-1.09	4.945E-02
ENSG00000250067	YJEFN3	YjeF N-terminal domain containing 3	-1.07	8.400E-06
ENSG00000145832	SLC25A48	solute carrier family 25 member 48	-1.06	5.218E-04
ENSG00000118997	DNAH7	dynein axonemal heavy chain 7	-1.05	1.032E-03
ENSG00000066923	STAG3	stromal antigen 3	-1.02	5.430E-09
ENSG00000213462	ERV3-1	endogenous retrovirus group 3 member 1, envelope	-1.02	5.190E-12
ENSG00000163126	ANKRD23	ankyrin repeat domain 23	-1.01	1.012E-02
ENSG00000009694	TENM1	teneurin transmembrane protein 1	-1	3.873E-03
ENSG00000118515	SGK1	serum/glucocorticoid regulated kinase 1	-0.99	4.003E-02
ENSG00000285053	TBCE	tubulin folding cofactor E	-0.99	2.930E-02
ENSG00000230551	AC021078.1	novel transcript	-0.98	6.540E-07
ENSG00000184206	GOLGA6L4	golgin A6 family like 4	-0.97	1.101E-03
ENSG00000262152	LINC00514	long intergenic non-protein coding RNA 514	-0.96	3.682E-02
ENSG00000123572	NRK	Nik related kinase	-0.96	1.822E-02

ENSG00000084674	APOB	apolipoprotein B	-0.95	1.550E-05
ENSG00000139537	CCDC65	coiled-coil domain containing 65	-0.94	4.764E-02
ENSG00000243708	PLA2G4B	phospholipase A2 group IVB	-0.94	4.376E-02
ENSG00000274808	TBC1D3B	TBC1 domain family member 3B	-0.94	4.315E-02
ENSG00000243696	AC006254.1	novel MUSTN1-ITIH4 readthrough	-0.94	4.741E-03
ENSG00000082196	C1QTNF3	C1q and TNF related 3	-0.93	3.840E-02
ENSG00000185864	NPIPB4	nuclear pore complex interacting protein family member B4	-0.93	3.730E-05
ENSG00000273154	AL121845.3	novel protein, ZGPAT-LIME1 readthrough	-0.92	1.613E-02
ENSG00000174501	ANKRD36C	ankyrin repeat domain 36C	-0.92	8.990E-05
ENSG00000181722	ZBTB20	zinc finger and BTB domain containing 20	-0.91	5.320E-06
ENSG00000213903	LTB4R	leukotriene B4 receptor	-0.89	3.267E-04
ENSG00000186166	CCDC84	coiled-coil domain containing 84	-0.89	1.926E-04
ENSG00000246922	UBAP1L	ubiquitin associated protein 1 like	-0.85	1.607E-02
ENSG00000114270	COL7A1	collagen type VII alpha 1 chain	-0.84	9.330E-06
ENSG00000139190	VAMP1	vesicle associated membrane protein 1	-0.83	4.285E-02
ENSG00000101104	PABPC1L	poly(A) binding protein cytoplasmic 1 like	-0.82	4.790E-10
ENSG00000114857	NKTR	natural killer cell triggering receptor	-0.82	7.110E-17
ENSG00000254995	STX16-NPEPL	STX16-NPEPL1 readthrough (NMD candidate)	-0.81	7.399E-03
ENSG00000173559	NABP1	nucleic acid binding protein 1	-0.8	4.802E-03
ENSG00000241489	AC244197.3	novel protein	-0.79	2.570E-08
ENSG00000175265	GOLGA8A	golgin A8 family member A	-0.78	1.700E-21
ENSG00000171811	CFAP46	cilia and flagella associated protein 46	-0.77	4.338E-02
ENSG00000198556	ZNF789	zinc finger protein 789	-0.76	1.498E-02
ENSG00000156042	CFAP70	cilia and flagella associated protein 70	-0.75	2.785E-02
ENSG00000169246	NPIPB3	nuclear pore complex interacting protein family member B3	-0.75	5.040E-10
ENSG00000204248	COL11A2	collagen type XI alpha 2 chain	-0.74	4.441E-02
ENSG00000197748	CFAP43	cilia and flagella associated protein 43	-0.74	3.905E-02
ENSG00000157765	SLC34A2	solute carrier family 34 member 2	-0.74	3.210E-02
ENSG00000160201	U2AF1	U2 small nuclear RNA auxiliary factor 1	-0.74	2.847E-02
ENSG00000278662	GOLGA6L10	golgin A6 family like 10	-0.74	2.098E-03
ENSG00000188738	FSIP2	fibrous sheath interacting protein 2	-0.74	1.958E-03
ENSG00000204172	AGAP9	ArfGAP with GTPase domain, ankyrin repeat and PH domain 9	-0.73	1.166E-02
ENSG00000206530	CFAP44	cilia and flagella associated protein 44	-0.72	2.485E-02
ENSG00000111664	GNB3	G protein subunit beta 3	-0.72	2.388E-02

ENSG00000135905	DOCK10	dedicator of cytokinesis 10	-0.7	2.069E-03
ENSG00000180626	ZNF594	zinc finger protein 594	-0.7	6.325E-04
ENSG00000062370	ZNF112	zinc finger protein 112	-0.69	4.198E-02
ENSG00000214029	ZNF891	zinc finger protein 891	-0.68	7.991E-04
ENSG00000166532	RIMKLB	ribosomal modification protein rimK like family member B	-0.68	2.570E-07
ENSG00000177990	DPY19L2	dpy-19 like 2	-0.67	4.304E-02
ENSG00000120327	PCDHB14	protocadherin beta 14	-0.67	3.487E-02
ENSG00000104894	CD37	CD37 molecule	-0.67	3.259E-02
ENSG00000185829	ARL17A	ADP ribosylation factor like GTPase 17A	-0.67	1.691E-02
ENSG00000182310	SPACA6	sperm acrosome associated 6	-0.67	4.149E-03
ENSG00000152926	ZNF117	zinc finger protein 117	-0.67	4.890E-08
ENSG00000128000	ZNF780B	zinc finger protein 780B	-0.66	3.376E-02
ENSG00000139631	CSAD	cysteine sulfinic acid decarboxylase	-0.66	3.869E-04
ENSG00000167615	LENG8	leukocyte receptor cluster member 8	-0.65	5.803E-04
ENSG00000163945	UVSSA	UV stimulated scaffold protein A	-0.65	2.430E-07
ENSG00000184619	KRBA2	KRAB-A domain containing 2	-0.64	2.575E-02
ENSG00000166436	TRIM66	tripartite motif containing 66	-0.64	2.060E-02
ENSG00000214021	TTLL3	tubulin tyrosine ligase like 3	-0.64	1.212E-02
ENSG00000145824	CXCL14	C-X-C motif chemokine ligand 14	-0.64	2.301E-04
ENSG00000275740	AC091959.3	novel readthrough transcript	-0.63	2.843E-02
ENSG00000172345	STARD5	StAR related lipid transfer domain containing 5	-0.62	1.872E-02
ENSG00000185513	L3MBTL1	L3MBTL histone methyl-lysine binding protein 1	-0.62	7.850E-07
ENSG00000204410	MSH5	mutS homolog 5	-0.6	2.388E-02
ENSG00000137474	MYO7A	myosin VIIA	-0.6	3.612E-03
ENSG00000186472	PCLO	piccolo presynaptic cytomatrix protein	-0.6	2.109E-03
ENSG00000167524	RSKR	ribosomal protein S6 kinase related	-0.6	6.001E-04
ENSG00000189367	KIAA0408	KIAA0408	-0.59	4.014E-02
ENSG00000196912	ANKRD36B	ankyrin repeat domain 36B	-0.58	4.044E-02
ENSG00000157423	HYDIN	HYDIN axonemal central pair apparatus protein	-0.58	6.143E-03
ENSG00000241404	EGFL8	EGF like domain multiple 8	-0.58	5.224E-03
ENSG00000130518	IQCN	IQ motif containing N	-0.57	4.409E-02
ENSG00000265590	C21orf59-TCP	CFAP298-TCP10L readthrough	-0.57	8.744E-03
ENSG00000104899	AMH	anti-Mullerian hormone	-0.56	1.959E-02
ENSG00000162825	NBPF20	NBPF member 20	-0.56	1.690E-02
ENSG00000162601	MYSM1	Myb like, SWIRM and MPN domains 1	-0.56	3.645E-03
ENSG00000127914	AKAP9	A-kinase anchoring protein 9	-0.56	1.821E-04
ENSG00000197978	GOLGA6L9	golgin A6 family like 9	-0.56	9.290E-07
ENSG00000243716	NPIPB5	nuclear pore complex interacting protein family member B5	-0.56	5.190E-12

ENSG00000154265	ABCA5	ATP binding cassette subfamily A member 5	-0.54	1.003E-02
ENSG00000121454	LHX4	LIM homeobox 4	-0.54	6.480E-03
ENSG00000156313	RPGR	retinitis pigmentosa GTPase regulator	-0.54	3.209E-03
ENSG00000197774	EME2	essential meiotic structure-specific endonuclease subunit 2	-0.54	2.133E-04
ENSG00000008311	AASS	aminoadipate-semialdehyde synthase	-0.53	4.338E-02
ENSG00000145476	CYP4V2	cytochrome P450 family 4 subfamily V member 2	-0.53	3.183E-02
ENSG00000137496	IL18BP	interleukin 18 binding protein	-0.53	1.256E-02
ENSG00000214193	SH3D21	SH3 domain containing 21	-0.53	3.635E-03
ENSG00000188234	AGAP4	ArfGAP with GTPase domain, ankyrin repeat and PH domain 4	-0.53	5.324E-04
ENSG00000163728	TTC14	tetratricopeptide repeat domain 14	-0.53	1.728E-04
ENSG00000126775	ATG14	autophagy related 14	-0.52	4.088E-03
ENSG00000054654	SYNE2	spectrin repeat containing nuclear envelope protein 2	-0.52	1.020E-03
ENSG00000116560	SFPQ	splicing factor proline and glutamine rich	-0.52	4.740E-06
ENSG00000137393	RNF144B	ring finger protein 144B	-0.51	4.696E-02
ENSG00000138658	ZGRF1	zinc finger GRF-type containing 1	-0.51	4.355E-02
ENSG00000080823	MOK	MOK protein kinase	-0.51	2.282E-02
ENSG00000059588	TARBP1	TAR (HIV-1) RNA binding protein 1	-0.51	1.865E-03
ENSG00000176155	CCDC57	coiled-coil domain containing 57	-0.51	2.522E-04
ENSG00000215788	TNFRSF25	TNF receptor superfamily member 25	-0.5	4.179E-02
ENSG00000166801	FAM111A	family with sequence similarity 111 member A	-0.5	2.584E-03
ENSG00000144749	LRIG1	leucine rich repeats and immunoglobulin like domains 1	-0.5	1.819E-03
ENSG00000055609	KMT2C	lysine methyltransferase 2C	-0.5	1.549E-04
ENSG00000198625	MDM4	MDM4 regulator of p53	-0.5	8.710E-05
ENSG00000138002	IFT172	intraflagellar transport 172	-0.49	3.576E-04
ENSG00000189042	ZNF567	zinc finger protein 567	-0.48	4.229E-02
ENSG00000232593	KANTR	KDM5C adjacent transcript	-0.48	1.073E-02
ENSG00000196876	SCN8A	sodium voltage-gated channel alpha subunit 8	-0.48	8.696E-03
ENSG00000121310	ECHDC2	enoyl-CoA hydratase domain containing 2	-0.48	4.149E-03
ENSG00000163660	CCNL1	cyclin L1	-0.48	1.600E-05
ENSG00000102908	NFAT5	nuclear factor of activated T cells 5	-0.48	3.980E-07
ENSG00000256591	AP003108.2	novel transcript	-0.47	4.691E-02
ENSG00000128849	CGNL1	cingulin like 1	-0.47	4.517E-02
ENSG00000160298	C21orf58	chromosome 21 open reading frame 58	-0.47	2.450E-02

ENSG00000008196	TFAP2B	transcription factor AP-2 beta	-0.47	2.001E-02
ENSG00000113916	BCL6	BCL6 transcription repressor	-0.47	1.233E-02
ENSG00000148143	ZNF462	zinc finger protein 462	-0.47	1.169E-02
ENSG00000233024	NPIPA9	nuclear pore complex interacting protein family, member A9	-0.47	5.997E-03
ENSG00000092094	OSGEP	O-sialoglycoprotein endopeptidase	-0.47	2.882E-04
ENSG00000108848	LUC7L3	LUC7 like 3 pre-mRNA splicing factor	-0.47	1.930E-07
ENSG00000224078	SNHG14	small nucleolar RNA host gene 14	-0.47	1.030E-07
ENSG00000131711	MAP1B	microtubule associated protein 1B	-0.47	4.570E-09
ENSG00000151914	DST	dystonin	-0.47	9.020E-11
ENSG00000184205	TSPYL2	TSPY like 2	-0.46	1.811E-02
ENSG00000273136	NBPF26	NBPF member 26	-0.46	8.527E-03
ENSG00000144026	ZNF514	zinc finger protein 514	-0.46	4.031E-03
ENSG00000175455	CCDC14	coiled-coil domain containing 14	-0.46	6.050E-07
ENSG00000165323	FAT3	FAT atypical cadherin 3	-0.45	2.927E-02
ENSG00000165359	INTS6L	integrator complex subunit 6 like	-0.45	2.785E-02
ENSG00000145362	ANK2	ankyrin 2	-0.45	2.778E-03
ENSG00000075826	SEC31B	SEC31 homolog B, COPII coat complex component	-0.45	1.818E-03
ENSG00000122417	ODF2L	outer dense fiber of sperm tails 2 like	-0.45	9.462E-04
ENSG00000119547	ONECUT2	one cut homeobox 2	-0.45	6.534E-04
ENSG00000164048	ZNF589	zinc finger protein 589	-0.45	3.072E-04
ENSG00000118058	KMT2A	lysine methyltransferase 2A	-0.45	2.522E-04
ENSG00000153914	SREK1	splicing regulatory glutamic acid and lysine rich protein 1	-0.45	4.030E-05
ENSG00000167548	KMT2D	lysine methyltransferase 2D	-0.45	1.930E-05
ENSG00000122515	ZMIZ2	zinc finger MIZ-type containing 2	-0.45	1.330E-06
ENSG00000100354	TNRC6B	trinucleotide repeat containing adaptor 6B	-0.44	3.376E-02
ENSG00000083097	DOP1A	DOP1 leucine zipper like protein A	-0.44	2.909E-02
ENSG00000266714	MYO15B	myosin XVB	-0.44	2.567E-02
ENSG00000169856	ONECUT1	one cut homeobox 1	-0.44	2.282E-02
ENSG00000162836	ACP6	acid phosphatase 6, lysophosphatidic	-0.44	1.794E-02
ENSG00000163482	STK36	serine/threonine kinase 36	-0.44	2.114E-03
ENSG00000198466	ZNF587	zinc finger protein 587	-0.44	1.499E-04
ENSG00000066739	ATG2B	autophagy related 2B	-0.44	7.370E-05
ENSG00000134884	ARGLU1	arginine and glutamate rich 1	-0.44	2.680E-05
ENSG00000109046	WSB1	WD repeat and SOCS box containing 1	-0.44	3.980E-07
ENSG00000179909	ZNF154	zinc finger protein 154	-0.43	1.043E-02
ENSG00000052126	PLEKHA5	pleckstrin homology domain containing A5	-0.43	8.109E-03
ENSG00000132424	PNISR	PNN interacting serine and arginine rich protein	-0.43	4.097E-03
ENSG00000119707	RBM25	RNA binding motif protein 25	-0.43	9.030E-07

ENSG00000164066	INTU	inturned planar cell polarity protein	-0.42	4.169E-02
ENSG00000185946	RNPC3	RNA binding region (RNP1, RRM) containing 3	-0.42	1.896E-02
ENSG00000147454	SLC25A37	solute carrier family 25 member 37	-0.42	2.877E-03
ENSG00000120437	ACAT2	acetyl-CoA acetyltransferase 2	-0.42	7.390E-05
ENSG00000141449	GREB1L	GREB1 like retinoic acid receptor coactivator	-0.42	8.420E-07
ENSG00000079134	THOC1	THO complex 1	-0.41	4.518E-02
ENSG00000143341	HMCN1	hemicentin 1	-0.41	2.762E-02
ENSG00000117620	SLC35A3	solute carrier family 35 member A3	-0.41	2.664E-02
ENSG00000148690	FRA10AC1	FRA10A associated CGG repeat 1	-0.41	8.002E-03
ENSG00000150893	FREM2	FRAS1 related extracellular matrix 2	-0.41	7.569E-03
ENSG00000149231	CCDC82	coiled-coil domain containing 82	-0.41	1.495E-03
ENSG00000164199	ADGRV1	adhesion G protein-coupled receptor V1	-0.41	4.009E-04
ENSG00000184465	WDR27	WD repeat domain 27	-0.41	7.730E-05
ENSG00000129003	VPS13C	vacuolar protein sorting 13 homolog C	-0.41	5.630E-06
ENSG00000127481	UBR4	ubiquitin protein ligase E3 component n-recognin 4	-0.41	3.780E-10
ENSG00000196705	ZNF431	zinc finger protein 431	-0.4	3.286E-02
ENSG00000204104	TRAF3IP1	TRAF3 interacting protein 1	-0.4	2.376E-02
ENSG00000131127	ZNF141	zinc finger protein 141	-0.4	2.180E-02
ENSG00000198185	ZNF334	zinc finger protein 334	-0.4	1.410E-02
ENSG00000138756	BMP2K	BMP2 inducible kinase	-0.4	8.696E-03
ENSG00000169045	HNRNPH1	heterogeneous nuclear ribonucleoprotein H1	-0.4	1.598E-03
ENSG00000159086	PAXBP1	PAX3 and PAX7 binding protein 1	-0.4	1.232E-03
ENSG00000160145	KALRN	kalirin RhoGEF kinase	-0.4	8.817E-04
ENSG00000112972	HMGCS1	3-hydroxy-3-methylglutaryl-CoA synthase 1	-0.4	6.160E-08
ENSG00000154760	SLFN13	schlafen family member 13	-0.39	4.730E-02
ENSG00000131914	LIN28A	lin-28 homolog A	-0.39	3.440E-02
ENSG00000184144	CNTN2	contactin 2	-0.39	2.679E-02
ENSG00000205517	RGL3	ral guanine nucleotide dissociation stimulator like 3	-0.39	1.975E-02
ENSG00000147421	HMBOX1	homeobox containing 1	-0.39	1.741E-02
ENSG00000154358	OBSCN	obscurin, cytoskeletal calmodulin and titin-interacting RhoGEF	-0.39	1.737E-02
ENSG00000135164	DMTF1	cyclin D binding myb like transcription factor 1	-0.39	1.680E-03
ENSG00000197969	VPS13A	vacuolar protein sorting 13 homolog A	-0.39	1.659E-03
ENSG00000137504	CREBZF	CREB/ATF bZIP transcription factor	-0.39	6.667E-04
ENSG00000146830	GIGYF1	GRB10 interacting GYF protein 1	-0.39	3.014E-04
ENSG00000161996	WDR90	WD repeat domain 90	-0.38	3.390E-02
ENSG00000285437	POLR2J3	RNA polymerase II subunit J3	-0.38	2.810E-02

ENSG00000074755	ZZEF1	zinc finger ZZ-type and EF-hand domain containing 1	-0.38	1.369E-02
ENSG00000173889	PHC3	polyhomeotic homolog 3	-0.38	8.479E-03
ENSG00000007392	LUC7L	LUC7 like	-0.38	3.777E-03
ENSG00000004777	ARHGAP33	Rho GTPase activating protein 33	-0.38	3.189E-03
ENSG00000116580	GON4L	gon-4 like	-0.38	2.208E-03
ENSG00000100941	PNN	pinin, desmosome associated protein	-0.38	1.675E-03
ENSG00000105443	CYTH2	cytohesin 2	-0.38	2.250E-05
ENSG00000196458	ZNF605	zinc finger protein 605	-0.37	3.883E-02
ENSG00000197961	ZNF121	zinc finger protein 121	-0.37	2.091E-02
ENSG00000099326	MZF1	myeloid zinc finger 1	-0.37	2.060E-02
ENSG00000165699	TSC1	TSC complex subunit 1	-0.37	1.967E-02
ENSG00000165115	KIF27	kinesin family member 27	-0.37	1.845E-02
ENSG00000258890	CEP95	centrosomal protein 95	-0.37	1.655E-02
ENSG00000085224	ATRX	ATRX chromatin remodeler	-0.37	3.984E-03
ENSG00000157796	WDR19	WD repeat domain 19	-0.37	8.908E-04
ENSG00000185222	TCEAL9	transcription elongation factor A like 9	-0.37	1.070E-05
ENSG00000104722	NEFM	neurofilament medium	-0.36	4.423E-02
ENSG00000126870	WDR60	WD repeat domain 60	-0.36	3.972E-02
ENSG00000197323	TRIM33	tripartite motif containing 33	-0.36	3.064E-02
ENSG00000188811	NHLRC3	NHL repeat containing 3	-0.36	2.902E-02
ENSG00000161010	MRNIP	MRN complex interacting protein	-0.36	1.775E-02
ENSG00000179240	GVQW3	GVQW motif containing 3	-0.36	1.717E-02
ENSG00000219481	NBPF1	NBPF member 1	-0.36	1.166E-02
ENSG00000005810	MYCBP2	MYC binding protein 2	-0.36	1.044E-02
ENSG00000100288	CHKB	choline kinase beta	-0.36	1.043E-02
ENSG00000173166	RAPH1	Ras association (RalGDS/AF-6) and pleckstrin homology domains 1	-0.36	8.437E-03
ENSG00000102901	CENPT	centromere protein T	-0.36	8.265E-03
ENSG00000117616	RSRP1	arginine and serine rich protein 1	-0.36	4.557E-03
ENSG00000104177	MYEF2	myelin expression factor 2	-0.36	2.189E-03
ENSG00000198707	CEP290	centrosomal protein 290	-0.36	1.675E-03
ENSG00000138688	KIAA1109	KIAA1109	-0.36	1.283E-03
ENSG00000184677	ZBTB40	zinc finger and BTB domain containing 40	-0.35	4.395E-02
ENSG00000152104	PTPN14	protein tyrosine phosphatase non-receptor type 14	-0.35	3.159E-02
ENSG00000117625	RCOR3	REST corepressor 3	-0.35	2.388E-02
ENSG00000197162	ZNF785	zinc finger protein 785	-0.35	2.205E-02
ENSG00000183426	NPIPA1	nuclear pore complex interacting protein family member A1	-0.35	1.875E-02
ENSG00000110888	CAPRIN2	caprin family member 2	-0.35	1.212E-02
ENSG00000047188	YTHDC2	YTH domain containing 2	-0.35	4.152E-03
ENSG00000109920	FNBP4	formin binding protein 4	-0.35	3.644E-03

ENSG00000122566	HNRNPA2B1	heterogeneous nuclear ribonucleoprotein A2/B1	-0.34	3.796E-02
ENSG00000139718	SETD1B	SET domain containing 1B, histone lysine methyltransferase	-0.34	3.608E-02
ENSG00000185658	BRWD1	bromodomain and WD repeat domain containing 1	-0.34	1.362E-02
ENSG00000167978	SRRM2	serine/arginine repetitive matrix 2	-0.34	1.266E-02
ENSG00000165434	PGM2L1	phosphoglucomutase 2 like 1	-0.34	1.097E-02
ENSG00000139910	NOVA1	NOVA alternative splicing regulator 1	-0.34	6.707E-03
ENSG00000080603	SRCAP	Snf2 related CREBBP activator protein	-0.34	9.462E-04
ENSG00000082438	COBLL1	cordon-bleu WH2 repeat protein like 1	-0.33	4.960E-02
ENSG00000096093	EFHC1	EF-hand domain containing 1	-0.33	4.764E-02
ENSG00000185246	PRPF39	pre-mRNA processing factor 39	-0.33	9.782E-03
ENSG00000112159	MDN1	midasin AAA ATPase 1	-0.33	9.012E-03
ENSG00000003402	CFLAR	CASP8 and FADD like apoptosis regulator	-0.33	3.645E-03
ENSG00000173230	GOLGB1	golgin B1	-0.33	1.675E-03
ENSG00000127603	MACF1	microtubule actin crosslinking factor 1	-0.33	1.821E-04
ENSG00000003756	RBM5	RNA binding motif protein 5	-0.33	2.250E-05
ENSG00000114127	XRN1	5'-3' exoribonuclease 1	-0.32	4.435E-02
ENSG00000079102	RUNX1T1	RUNX1 partner transcriptional co-repressor 1	-0.32	4.011E-02
ENSG00000196440	ARMCX4	armadillo repeat containing X-linked 4	-0.32	2.403E-02
ENSG00000166987	MBD6	methyl-CpG binding domain protein 6	-0.32	3.582E-03
ENSG00000123636	BAZ2B	bromodomain adjacent to zinc finger domain 2B	-0.32	2.603E-03
ENSG00000151150	ANK3	ankyrin 3	-0.32	3.395E-04
ENSG00000011021	CLCN6	chloride voltage-gated channel 6	-0.31	3.959E-02
ENSG00000150593	PDCD4	programmed cell death 4	-0.31	3.786E-02
ENSG00000115977	AAK1	AP2 associated kinase 1	-0.31	2.664E-02
ENSG00000089335	ZNF302	zinc finger protein 302	-0.31	1.256E-02
ENSG00000136944	LMX1B	LIM homeobox transcription factor 1 beta	-0.31	1.044E-02
ENSG00000090924	PLEKHG2	pleckstrin homology and RhoGEF domain containing G2	-0.31	1.003E-02
ENSG00000067064	IDI1	isopentenyl-diphosphate delta isomerase 1	-0.31	8.696E-03
ENSG00000131437	KIF3A	kinesin family member 3A	-0.31	4.149E-03
ENSG00000116754	SRSF11	serine and arginine rich splicing factor 11	-0.31	2.726E-03
ENSG00000221978	CCNL2	cyclin L2	-0.31	4.040E-05

ENSG00000177200	CHD9	chromodomain helicase DNA binding protein 9	-0.3	4.738E-02
ENSG00000048707	VPS13D	vacuolar protein sorting 13 homolog D	-0.3	4.169E-02
ENSG00000115414	FN1	fibronectin 1	-0.3	3.786E-02
ENSG00000078687	TNRC6C	trinucleotide repeat containing adaptor 6C	-0.3	3.128E-02
ENSG00000137343	ATAT1	alpha tubulin acetyltransferase 1	-0.3	3.094E-02
ENSG00000157741	UBN2	ubinuclein 2	-0.3	3.005E-02
ENSG00000134909	ARHGAP32	Rho GTPase activating protein 32	-0.3	1.410E-02
ENSG00000171316	CHD7	chromodomain helicase DNA binding protein 7	-0.3	1.266E-02
ENSG00000164050	PLXNB1	plexin B1	-0.3	4.498E-03
ENSG00000131051	RBM39	RNA binding motif protein 39	-0.3	3.189E-03
ENSG00000148798	INA	internexin neuronal intermediate filament protein alpha	-0.3	2.952E-04
ENSG00000130396	AFDN	afadin, adherens junction formation factor	-0.3	2.301E-04
ENSG00000131013	PPIL4	peptidylprolyl isomerase like 4	-0.29	2.746E-02
ENSG00000134899	ERCC5	ERCC excision repair 5, endonuclease	-0.29	2.403E-02
ENSG00000197603	CPLANE1	ciliogenesis and planar polarity effector 1	-0.29	2.210E-02
ENSG00000173575	CHD2	chromodomain helicase DNA binding protein 2	-0.29	1.542E-02
ENSG00000160285	LSS	lanosterol synthase	-0.29	1.043E-02
ENSG00000113161	HMGCR	3-hydroxy-3-methylglutaryl-CoA reductase	-0.29	5.084E-03
ENSG00000103657	HERC1	HECT and RLD domain containing E3 ubiquitin protein ligase family member 1	-0.29	2.189E-03
ENSG00000119596	YLPM1	YLP motif containing 1	-0.29	1.057E-03
ENSG00000147162	OGT	O-linked N-acetylglucosamine (GlcNAc) transferase	-0.29	2.849E-04
ENSG00000143624	INTS3	integrator complex subunit 3	-0.29	2.841E-04
ENSG00000100201	DDX17	DEAD-box helicase 17	-0.29	6.230E-05
ENSG00000106344	RBM28	RNA binding motif protein 28	-0.28	4.050E-02
ENSG00000099194	SCD	stearoyl-CoA desaturase	-0.28	3.943E-02
ENSG00000109756	RAPGEF2	Rap guanine nucleotide exchange factor 2	-0.28	3.440E-02
ENSG00000151320	AKAP6	A-kinase anchoring protein 6	-0.28	1.896E-02
ENSG00000189180	ZNF33A	zinc finger protein 33A	-0.28	1.674E-02
ENSG00000125844	RRBP1	ribosome binding protein 1	-0.28	1.561E-02
ENSG00000086758	HUWE1	HECT, UBA and WWE domain containing E3 ubiquitin protein ligase 1	-0.28	1.185E-02
ENSG00000123384	LRP1	LDL receptor related protein 1	-0.28	5.599E-03

ENSG00000047410	TPR	translocated promoter region, nuclear basket protein	-0.28	4.498E-03
ENSG00000152795	HNRNPDL	heterogeneous nuclear ribonucleoprotein D like	-0.28	3.957E-03
ENSG00000074356	NCBP3	nuclear cap binding subunit 3	-0.28	2.726E-03
ENSG00000167522	ANKRD11	ankyrin repeat domain 11	-0.28	2.457E-03
ENSG00000158321	AUTS2	activator of transcription and developmental regulator AUTS2	-0.28	2.457E-03
ENSG00000107929	LARP4B	La ribonucleoprotein 4B	-0.28	2.217E-03
ENSG00000083857	FAT1	FAT atypical cadherin 1	-0.28	2.803E-04
ENSG00000176444	CLK2	CDC like kinase 2	-0.27	3.134E-02
ENSG00000013573	DDX11	DEAD/H-box helicase 11	-0.27	2.575E-02
ENSG00000134186	PRPF38B	pre-mRNA processing factor 38B	-0.27	2.500E-02
ENSG00000109654	TRIM2	tripartite motif containing 2	-0.27	2.372E-02
ENSG00000100393	EP300	E1A binding protein p300	-0.27	1.811E-02
ENSG00000137776	SLTM	SAFB like transcription modulator	-0.27	1.655E-02
ENSG00000001630	CYP51A1	cytochrome P450 family 51 subfamily A member 1	-0.27	1.003E-02
ENSG00000130164	LDLR	low density lipoprotein receptor	-0.27	8.848E-03
ENSG00000120549	KIAA1217	KIAA1217	-0.27	5.500E-03
ENSG00000123200	ZC3H13	zinc finger CCCH-type containing 13	-0.27	1.818E-03
ENSG00000173064	HECTD4	HECT domain E3 ubiquitin protein ligase 4	-0.27	6.964E-04
ENSG00000176953	NFATC2IP	nuclear factor of activated T cells 2 interacting protein	-0.26	4.549E-02
ENSG00000121390	PSPC1	paraspeckle component 1	-0.26	2.800E-02
ENSG00000172915	NBEA	neurobeachin	-0.26	2.741E-02
ENSG00000170004	CHD3	chromodomain helicase DNA binding protein 3	-0.26	2.510E-02
ENSG00000119760	SUPT7L	SPT7 like, STAGA complex subunit gamma	-0.26	2.455E-02
ENSG00000118418	HMGN3	high mobility group nucleosomal binding domain 3	-0.26	1.691E-02
ENSG00000197535	MYO5A	myosin VA	-0.26	1.627E-02
ENSG00000133858	ZFC3H1	zinc finger C3H1-type containing	-0.26	1.483E-02
ENSG00000038219	BOD1L1	biorientation of chromosomes in cell division 1 like 1	-0.26	1.034E-02
ENSG00000128731	HERC2	HECT and RLD domain containing E3 ubiquitin protein ligase 2	-0.26	1.011E-02
ENSG00000117523	PRRC2C	proline rich coiled-coil 2C	-0.26	4.438E-04
ENSG00000124177	CHD6	chromodomain helicase DNA binding protein 6	-0.25	4.975E-02
ENSG00000139613	SMARCC2	SWI/SNF related, matrix associated, actin dependent regulator of chromatin subfamily c member 2	-0.25	4.169E-02
ENSG00000111011	RSRC2	arginine and serine rich coiled-coil 2	-0.25	3.783E-02

ENSG00000119688	ABCD4	ATP binding cassette subfamily D member 4	-0.25	3.322E-02
ENSG00000140836	ZFHX3	zinc finger homeobox 3	-0.25	3.128E-02
ENSG00000197102	DYNC1H1	dynein cytoplasmic 1 heavy chain 1	-0.25	3.893E-04
ENSG00000196220	SRGAP3	SLIT-ROBO Rho GTPase activating protein 3	-0.24	4.599E-02
ENSG00000197119	SLC25A29	solute carrier family 25 member 29	-0.24	3.279E-02
ENSG00000107863	ARHGAP21	Rho GTPase activating protein 21	-0.24	3.101E-02
ENSG00000104549	SQLE	squalene epoxidase	-0.24	2.927E-02
ENSG00000168916	ZNF608	zinc finger protein 608	-0.24	1.549E-02
ENSG00000181222	POLR2A	RNA polymerase II subunit A	-0.24	1.044E-02
ENSG00000075292	ZNF638	zinc finger protein 638	-0.23	3.881E-02
ENSG00000005483	KMT2E	lysine methyltransferase 2E (inactive)	-0.22	4.365E-02
ENSG00000160752	FDPS	farnesyl diphosphate synthase	-0.22	4.362E-02
ENSG00000118482	PHF3	PHD finger protein 3	-0.22	4.227E-02
ENSG00000155368	DBI	diazepam binding inhibitor, acyl-CoA binding protein	-0.22	3.959E-02
ENSG00000198408	OGA	O-GlcNAcase	-0.22	3.896E-02
ENSG00000064607	SUGP2	SURP and G-patch domain containing 2	-0.22	3.591E-02
ENSG00000151835	SACS	sacsin molecular chaperone	-0.21	4.449E-02
ENSG00000117713	ARID1A	AT-rich interaction domain 1A	-0.21	3.769E-02
ENSG00000132780	NASP	nuclear autoantigenic sperm protein	-0.21	3.036E-02
ENSG00000054523	KIF1B	kinesin family member 1B	-0.2	2.111E-02
ENSG00000115306	SPTBN1	spectrin beta, non-erythrocytic 1	-0.2	1.699E-02
ENSG00000111605	CPSF6	cleavage and polyadenylation specific factor 6	-0.19	3.633E-02

List of differently expressed genes comparing mock and SARS-CoV-2 infected iPSC-derived pancreatic cells at Day 3 post infection with adjusted P value < 0.05
